# A Review of Alpha‑2 Adrenergic Agents Implicated in Opioid Use Disorder

**DOI:** 10.1021/acschemneuro.5c00970

**Published:** 2026-02-18

**Authors:** Audrey Hannum, Isabella Spano, Edward Ofori

**Affiliations:** Ohio Northern University Department of Pharmaceutical and Biomedical Sciences, Rudolph H. Raabe College of Pharmacy, 1044 Ohio Northern University, Ada, Ohio 45810, United States

**Keywords:** opioid use disorder, OUD, adrenergic, alpha-2 adrenergic agonist, xylazine, medetomidine, clonidine, opioid overdose, α_2_ agonist toxicity, public health

## Abstract

Opioid use disorder (OUD) has been recognized for many years as a leading public health emergency in the United States, costing almost 80,000 Americans their lives in 2023. Recently, fentanyl, the predominant synthetic opioid implicated in OUD, has begun to be adulterated with the alpha-2 adrenergic receptor (α_2_AR) agonists xylazine and medetomidine. This has a profound effect on the treatment of suspected overdoses, as naloxone, which reverses fentanyl overdoses, does not reverse α_2_ agonist overdoses, and there are no FDA-approved treatments for overdoses with an α_2_ agonist. Xylazine is spreading rapidly through the illicit drug supply in the United States, and to further complicate efforts to mitigate its effects, xylazine users frequently experience necrotic wounds which are difficult to treat. In this review, we comprehensively examined literature sources to compile information on the pharmacology, chemistry, pharmacokinetics, and toxicities of α_2_AR agonists that are implicated in OUD. We hope that this summary will provide a perspective on the way forward in combating the issue of α_2_ agonist-adulterated opioids and lead to the discovery of drugs as reversal agents for α_2_ agonist overdoses.

## Graphical abstract

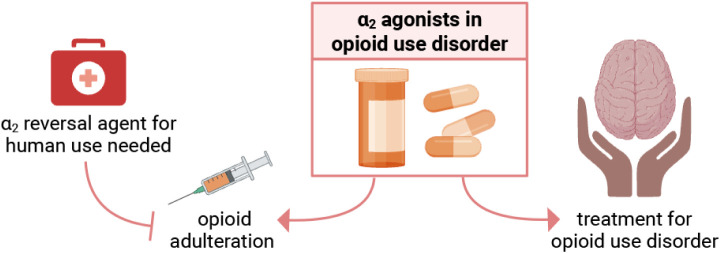

## Introduction

Opioid use disorder (OUD) is a chronic condition characterized by the compulsive use of opioids despite significant distress or impairment, including fatal overdoses. It remains a critical public health crisis globally, with a particularly severe impact in the United States. In 2021, an estimated 6.1 million individuals aged 12 or older were affected by OUD in the U.S.^1^

Current treatment strategies for OUD include medications for opioid use disorder (MOUD), such as buprenorphine,^2^ methadone,^3^ and naltrexone.^4^ The opioid epidemic has been exacerbated by the emergence of synthetic opioids like fentanyl, which are far more potent and lethal than traditional opioids. Recently, alpha-2 adrenergic receptor (α_2_AR) agonists, such as xylazine **(1)** and medetomidine **(2)**, have gained attention as they increasingly appear in the illicit drug supply, particularly in combination with opioids such as heroin and fentanyl.^5^ The DEA reports that xylazine and fentanyl mixtures have been found in 48 of 50 states in the U.S. as of 2022 and among those confiscated drugs, approximately 23% of fentanyl powder and 7% of fentanyl pills contained xylazine.^6^ This suggests a strong ecological association of fentanyl and xylazine.^7^

A comprehensive study conducted in Philadelphia, the epicenter of the xylazine epidemic, revealed a staggering increase from 2% to 26% in drug overdose deaths involving xylazine within a five-year period from 2015 to 2020.^8^ The increasing prevalence of opioid adulteration with α_2_ agonists has introduced new challenges in overdose management. These α_2_ agonists exacerbate opioid-induced respiratory depression and complicate emergency medical interventions due to the absence of clinically approved reversing agents.

This review focuses on the pharmacology, chemistry, pharmacokinetics and public health impact of α_2_ adrenergic drugs implicated in OUD (Figure 1). Certain α_2_ adrenergic drugs are known adulterants of fentanyl, namely xylazine and medetomidine, while others have been studied for the treatment of opioid withdrawal. To this end, we researched through the National Library of Medicine, Google Scholar and Patents, using keywords that included: Opioid Use Disorder, OUD, adrenergic, alpha-2 adrenergic agonist, xylazine, medetomidine, clonidine, public health, opioid overdose, α_2_ agonist toxicity.

**Figure 1:**
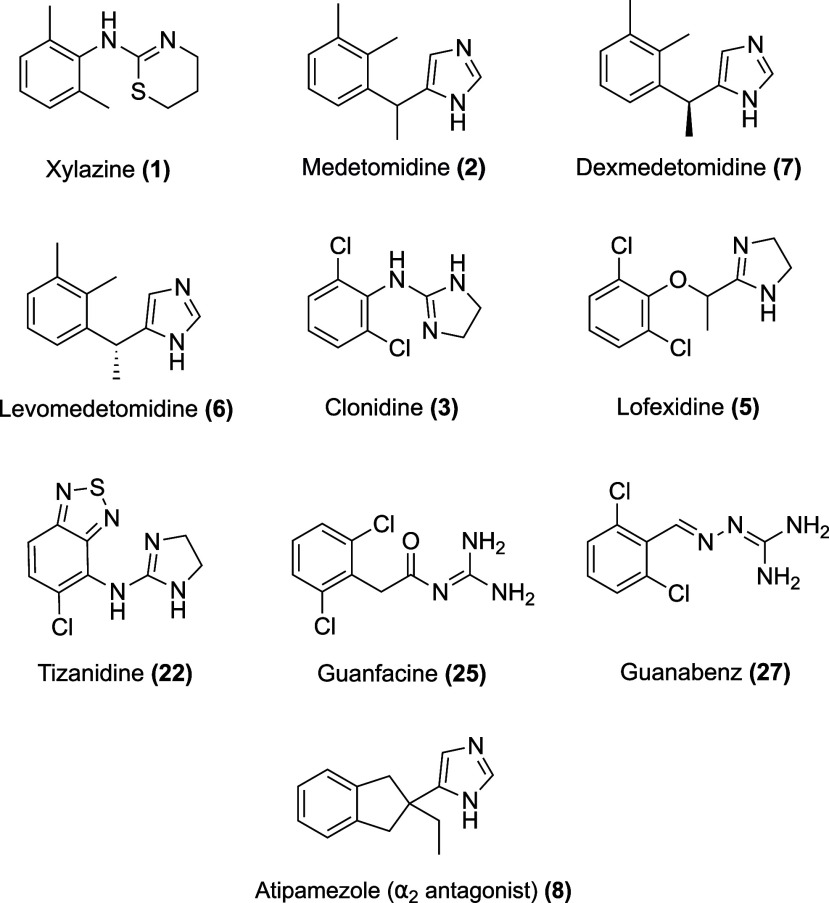
Structures of selected FDA-approved α_2_ agonists and antagonists.

## Characteristics of Selected α_2_ Agents Implicated in OUD

### Xylazine

#### History

Xylazine **(1)** (*Rompun*, *Anased*, *Sedazine*) is a veterinary sedative used for procedural anesthesia in dogs, cats, horses, and deer.^9^ Xylazine first emerged in Puerto Rico in the early 2000s as an adulterant of speedball, or cocaine mixed with heroin.^10^ Xylazine is added to opioids to extend the high that they produce, since its sedative properties lengthen the effects of the opioid.^11^ While it is not clear how xylazine first became popular in the drug market, limited evidence suggests that it first emerged in rural ranching communities, suggesting that users had access to veterinary supplies of xylazine.^10^ Prior to 2006, there were no reported cases of xylazine use in Philadelphia;^12^ then, by the 2010s, xylazine started to be found in the city’s drug supply.^8^ At the time, the Puerto Rican government was relocating drug users from Puerto Rico to the United States because there were few addiction treatment centers in Puerto Rico, and Philadelphia was a major target of these relocations. However, the treatment centers lacked knowledge regarding xylazine use and did not regulate it among their patients, allowing a demand for xylazine and a source of xylazine to spread. More recently, xylazine supplies remain concentrated in Puerto Rican neighborhoods in Philadelphia, with open-air markets in these neighborhoods being a common source of xylazine-laced drugs. Drug dealers may be linked between the two locations, allowing for a ready demand and source of xylazine.^8^ Since its introduction to Philadelphia, xylazine has spread throughout the United States, although it remains most prevalent in the Northeast.^13^

#### Demographics

Today, xylazine is mainly found as an adulterant in fentanyl, as fentanyl has largely replaced heroin as the opioid of choice. Demographic data from overdose deaths from heroin or fentanyl in Philadelphia between 2010 and 2019 revealed that xylazine users were generally white men between the ages 35 and 54.^14^ Similar results were found among decedents from overdose deaths between 2019 and 2023 in Michigan.^15^ A survey of 43,947 American adults assessed for substance use treatment found that xylazine users had more nonfatal overdoses on average than nonxylazine users. It is not known if this indicates some protective effect of xylazine, whereby users are likely to have more overdoses but are more likely to survive them, or if both increased nonfatal overdoses and xylazine use are simply correlates of polysubstance use.^16^

#### Pharmacology

Xylazine is a partial agonist of α_2_ adrenergic receptors, decreasing the release of norepinephrine.^17,18^ Xylazine has a markedly lower binding affinity and potency at α_2_ARs; for example, clonidine is has approximately a 93-times higher binding affinity at the α_2A_ receptor than xylazine^19^ (Table 1). While one study in mice found that the α_2A_ receptor subtype may play a key role in xylazine’s analgesic and sedative effects,^20^ other research has not supported this subtype specificity and has found an equal affinity for the receptor subtypes.^21^ Animal models have shown that xylazine induces the release of endogenous opioids that bind mu opioid receptors, but not delta and kappa opioid receptors. This is responsible for xylazine’s antinociceptive effects^22^ (Figure 2). New research also suggests that xylazine is itself an agonist for kappa opioid receptors, although this result has not been replicated in humans.^23^

**Table 1:** Binding Affinity and Potency of α_2_ Agonists at α_2A_, α_2B_, and α_2C_ Adrenergic Receptors

	Binding affinity (p*K*_i_)			Potency (pEC_50_)		
	α_2A_	α_2B_	α_2C_	α_2A_	α_2B_	α_2C_
Xylazine^19,21^	5.24	5.46	4.81	5.73	5.72	5.46
Dexmedetomidine^19^	7.88	7.47	7.02	8.50	8.48	7.54
Medetomidine^21,27^	9.59	9.87	10.09	7.13–9.68	9.19–9.69	9.71
Clonidine^19^	7.21	7.16	6.87	7.57	7.25	6.04
Lofexidine^120,183^	9.86	n/a	10.08	9.1	7.9	8.5
Tizanidine^27,184^	10.75	11.25	11.08	5.8–8.4	6.3–7	6.82
Guanfacine^19^	7.03	5.86	5.41	7.28	6.54	6.22
Guanabenz^19^	7.66	6.55	6.35	8.25	7.01	<5

**Figure 2:**
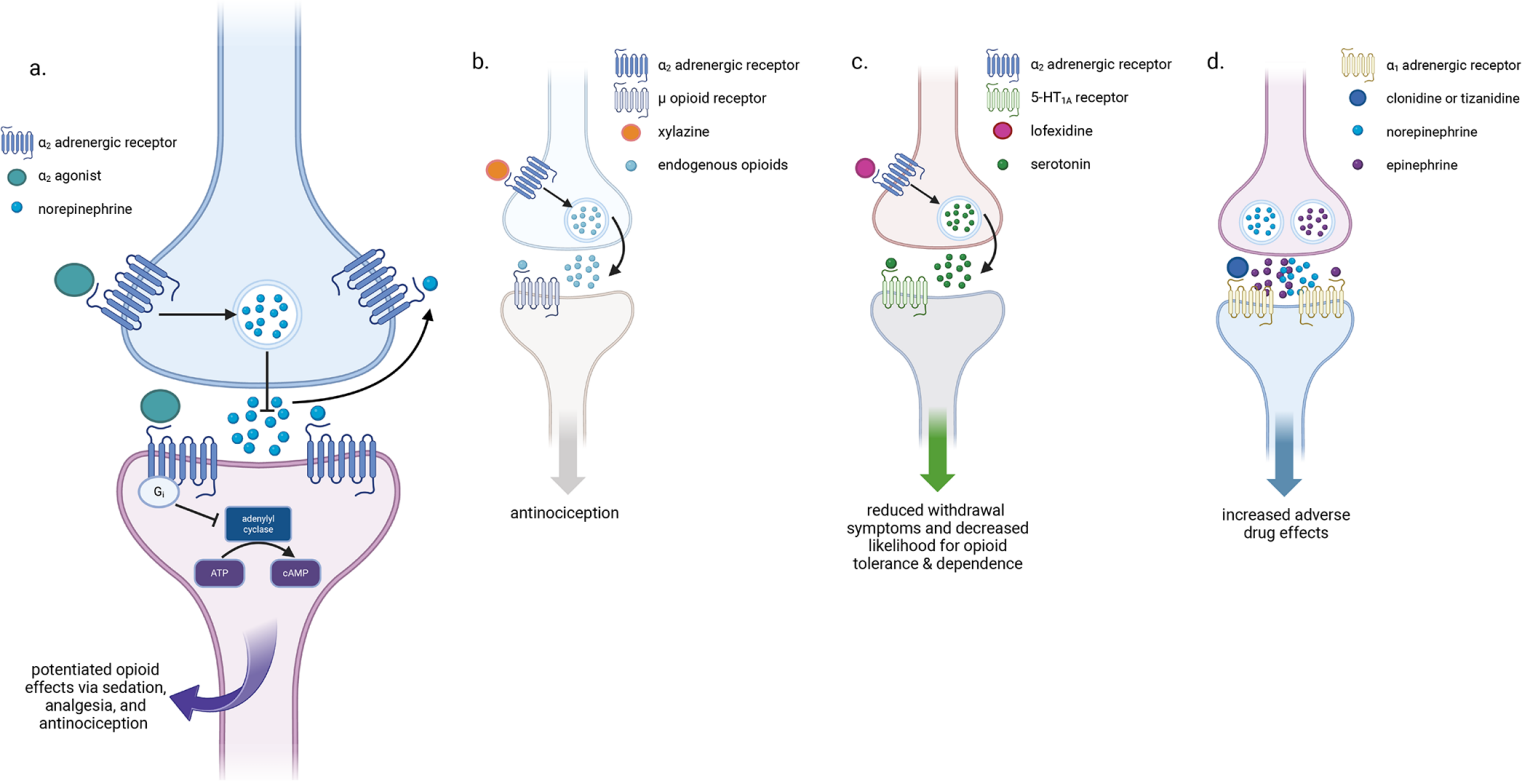
Mechanism of action of selected α_2_ agents in signaling pathways in the central nervous system implicated in OUD. (a) α_2_ agonists at the α_2_ adrenergic receptor. (b) Xylazine **(1)** crosstalk with μ opioid receptor. (c) Lofexidine **(5)** crosstalk with 5-HT_1A_ receptor. (d) Clonidine **(3)** and tizanidine **(22)** at the α_1_ adrenergic receptor.

The α_2_AR is coupled to the G_i/o_ G-protein, which upon activation inhibits adenylyl cyclase and voltage gated calcium channels, and opens potassium channels (Figure 2). However, α_2_ agonists can also activate G_s_ signaling pathways, as evidenced by the identification of distinct binding domains in the α_2A_ adrenergic receptor for G_s_ versus G_i_ coupling.^19,24^ It is hypothesized that α_2_ agonists may act on G_s_ via Gβγ, which is released from Gα_i_ and activates adenylyl cyclase in a synergistic effect with the direct activation of adenylyl cyclase by G_s_.^25,26^

Like other α_2_ agonists, xylazine activates G_i_ at low doses, but activates G_s_ at higher doses, albeit exhibiting a weaker G_s_-coupling response than other α_2_ agonists such as brimonidine.^27^ As drugs that activate the G_s_ pathway selectively have fewer side effects, such as sedation,^28^ G_s_-biased agonists are an exciting target of current drug discovery efforts that seek to minimize adverse reactions.

#### Effects

As an α_2_ agonist, xylazine lowers body temperature and blood pressure and has a sedating effect.^29^ Xylazine also decreases blood oxygen levels in the brain, partly due to slowing ventilation, although vasoconstriction of cerebral vessels may have an independent effect.^29,30^ Fentanyl, as an opioid, also lowers blood oxygen levels in the brain by depressing the respiratory rate; however, this response is temporary, and brain oxygen levels gradually rebound. When xylazine and fentanyl are combined, the brain oxygen level rebound is suppressed, leading to a longer period of hypoxia.^29^ In mice, coadministration of xylazine and fentanyl was shown to lower respiratory rate more than fentanyl alone. Specifically, xylazine extends expiratory time when combined with fentanyl, which reduces inspiratory time.^31^ Long-term xylazine use has also been reported to result in cardiac necrosis, cardiac fibrosis, lung edema, and lung congestion.^32^

#### Toxicology

There is not a straightforward way to differentiate between fatal and nonfatal doses of xylazine. Reported cases of xylazine toxicity and fatality present xylazine doses of between 400 and 2,400 mg. Other reports of nonfatal cases present doses that overlap with this range. This underscores the potential dangers of xylazine use, since there is no dose of xylazine that can be considered safe.^33^

Xylazine overdose is not resolved by treatment with naloxone, since xylazine is not an opioid. While mice in the study demonstrating xylazine’s kappa opioid agonist activity did respond to naloxone treatment, this effect is not generally seen in humans.^23^ Limited data suggest that naloxone may be efficacious in the treatment of clonidine toxicity in children when given at sufficient doses. Specifically, naloxone treatment was found to be effective in reversing sedation and bradycardia caused by this α_2_ agonist. The suggested mechanism is that clonidine triggers the release of β-endorphin, an endogenous opiate, especially in cases of clonidine overdose.^34^ However, these results are debated,^35^ and while xylazine and clonidine are similar in structure, it is not known whether opioid release is also secondary to xylazine use.

#### Wounds

One of the most dangerous effects of xylazine use is the formation of xylazine-associated wounds. These wounds are typically found in the extremities and progress from a small blisters to necrotic ulcers that can eat away muscle and bone.^36,37^ Wounds frequently occur in drug users who inject xylazine, but are also common in users who inhale xylazine.^37^ While the pathophysiology of xylazine-associated wounds is not fully understood, it is hypothesized that xylazine’s α_2_ agonist activity leads to vasoconstriction of cutaneous vessels.^37,38^ Studies in animal models have shown that xylazine decreases local PO_2_ at the skin^39^ and increases blood glucose levels,^18,40^ both of which would impair wound healing.

In people who use drugs (PWUD), several other factors contribute to poor wound healing, allowing for the development of severe wounds. First, xylazine acts as a local analgesic, so users do not experience pain upon further injections, encouraging continued use.^14,37^ Additionally, wounds may be insensate, hence the finding that xylazine users frequently inject into the wound or around its edge to achieve rapid absorption of the drug.^41^ A recent study in Massachusetts found that xylazine-associated wounds were more common in drug users who practiced skin popping,^42^ or subcutaneous injection of the drug to achieve slower absorption, leading to less of a rush and a longer high.^43^ Finally, surveys of PWUD have shown that users prefer to care for their own wounds, with practices such as lancing the wound or using drug syringes to extract pus from the wound.

Many xylazine users with wounds avoid interacting with the healthcare system due to the stigma surrounding their drug use, in addition to the stigma of having an open wound, and many report that their withdrawal symptoms are inadequately managed during inpatient stays. In addition, patients with painful wounds are often denied pain medications because of their history of drug use. For this reason, xylazine users with wounds frequently choose to self-treat, or if they do seek help, it is often from their local syringe services program. These programs have become all too familiar with treating xylazine-induced wounds, but they are often understaffed and underfunded. Among PWUD surveyed in Massachusetts, over one in five reported being turned away from a rehabilitation program due to xylazine-related wounds.^42^ In all, xylazine-associated wounds represent a serious health threat to drug users that requires local treatment centers to become familiar with best practices for the care of these wounds.

Recommendations on xylazine-associated wound care from institutional practices in Philadelphia center on the importance of a multidisciplinary approach, bringing together peer navigators, wound care experts, addiction treatment specialists, and infectious disease physicians. For basic wound care, the framework the Philadelphia clinics have developed advises cleaning the wound with a nonirritating solution; applying a primary dressing that will keep the wound moist, as well as an autolytic or enzymatic product if the wound contains dead tissue; adding a secondary dressing to absorb drainage; and securing the wound covering with a reinforcing dressing that does not compress the wound. However, there is a great deal of nuance in treating xylazine-associated wounds, as decisions must be made about how much necrotic tissue to remove to prevent further infection while preserving islands of healthy tissue within the wound, and about which antibiotics to choose to address soft tissue infection. PWUD with xylazine-associated wounds must also have their other needs met, such as the need for addiction treatment to allow the patient to stop continually injecting xylazine, or needs for housing and access to continuing wound care.^44^

#### Pharmacokinetics

Due to its lipophilic nature, xylazine is widely and rapidly distributed, with a volume of distribution of 1.9 to 2.5 L/kg in animals.^33^ The half-life of xylazine in humans was calculated at 4.9 h from a single case of xylazine intoxication in a human patient.^45^ In animals, the elimination half-life of xylazine varies from 22 min in sheep to 50 min in horses,^46^ 4.5 h in rats,^47^ and 1.3 h in rats dosed with ketamine and xylazine.^48^ In humans, symptoms of xylazine may last from 8 to 72 h.^49^ Xylazine undergoes extensive metabolism before being excreted by the kidneys, with 2,6-dimethylaniline **(4)**, also known as 2,6-xylidine, being the dominant metabolic product of the breakdown of the thiazine ring.^50–52^ The metabolic pathway also includes *N*-dealkylation, hydroxylation, dealkylation, and *S*-oxidation^51^ (Figure 3). In rats, CYP3A has been shown to be responsible for metabolizing xylazine, but the CYP enzyme implicated in xylazine metabolism in humans has not yet been determined.^53^

**Figure 3:**
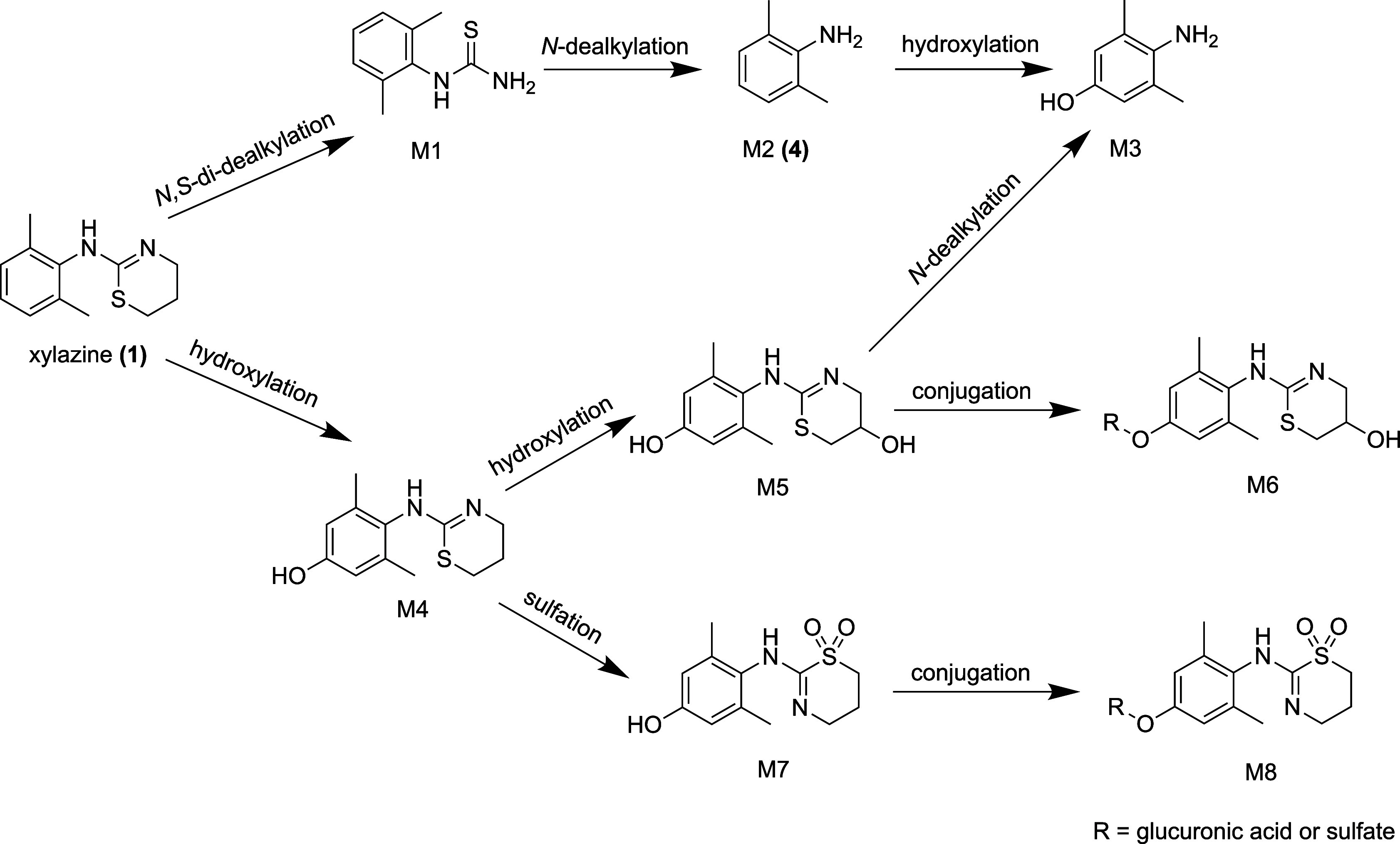
Metabolites of xylazine in human urine. A selection of possible transformations are shown, but similar transformations could also be observed at other positions (adapted from Meyer and Maurer^51^). M1: 2,6-dimethylphenylthiourea. M2: 2,6-dimethylaniline **(4)**. M3: 4-amino-3,5-dimethylphenol. M4: 4-hydroxy-xylazine. M5: 2-((4-hydroxy-2,6-dimethylphenyl)­amino)-5,6-dihydro-4*H*-1,3-thiazin-5-ol. M6: Conjugated metabolite of M5. M7: Sulfone metabolite of 4-hydroxy-xylazine. M8: Conjugated metabolite of M7.

#### Physicochemical Description

The chemical name of xylazine is *N*-(2,6-dimethylphenyl)-5,6-dihydro-4*H*-1,3-thiazin-2-amine. It is practically insoluble in water, but is soluble in methanol, ethanol, organic solvents, and dilute HCl. The solubility in ethanol, methanol and DMSO are 20 mg/mL, 50 mg/mL, and 30 mg/mL respectively.^54,55^ Xylazine HCl is a crystalline solid (Table 2).

**Table 2:** Physicochemical Properties of α_2_ Agonists^a^

	Melting point (°C)	Water solubility (mM)	cLog*P*	Molecular weight (g/mol)	PSA (Å^2^)
Xylazine HCl	136–139 ^55^	100–175 ^185,186^	2.92	256.80	49.69
Medetomidine HCl	151–153 ^187^	100 ^188^	2.88	236.74	28.68
Dexmedetomidine HCl	158 ^189^	100 ^190^	2.90	236.74	28.68
Clonidine HCl	130	100 ^191^	2.07	266.55	36.42
Lofexidine HCl	221–223	68 ^192^	2.71	295.59	33.62
Tizanidine HCl	280	52 ^193^	1.91	290.17	90.44
Guanfacine HCl	213–216	4 ^154^	1.83	282.55	81.47
Guanabenz acetate	225–227	17 ^194^	1.84	291.13	76.76

a cLog*P* = calculated log*P*_o/w_; PSA =polar surface area. Melting point data from DrugBank unless otherwise noted.Data on cLog*P*, molecular weight, and PSA from SwissADME.

In one study, clonidine, xylazine, and lofexidine **(5)**, an α_2_ agonist which is used to treat opioid withdrawal, were compared. The binding dissociation constant *K*_i_ was evaluated via an assay on rat cerebral cortex homogenate with the drugs of interest as inhibitors of *p*-amino clonidine. The *K*_i_ for clonidine, xylazine, and lofexidine were found to be 2.7 nM, 120 nM, and 2.3 nM respectively. The lower binding affinity for xylazine was associated with weaker hypotensive activity. The log*D*_7.4_ was 0.93 for clonidine and xylazine and 0.47 for lofexidine. This result explains the extensive metabolism of these compounds. Finally, the p*K*_a_ clonidine was found to be 8.05; for xylazine, 7.20; and for lofexidine, 8.90.^56^

## Medetomidine

### History and Demographics

Like xylazine, medetomidine **(2)** is a veterinary sedative, generally used in small animal practice.^57^ It was developed and marketed in Europe in 1987, and was first approved by the FDA in 1996 for use in dogs.^5^ Medetomidine was first detected in Maryland in July 2022 and was then found in cases of opioid overdose in Missouri, Colorado, Pennsylvania, California, and Maryland between 2022 and 2023.^58^ Medetomidine was found in Maryland as a result of the state’s Rapid Analysis of Drugs program, a public health initiative where law enforcement and public health workers collect samples from drug paraphernalia and send them to a lab for testing.^59,60^ In the fourth quarter of 2024 alone, medetomidine was found in 8.51% of the 282 samples collected by the program.^61^ The drug was first detected in Philadelphia and Pittsburgh in April and May of 2024, respectively, and in Chicago in May of 2024.^58^ In these three cities, medetomidine use was associated with recent outbreaks of opioid overdoses, and may have had a causative role.^62^ To date, medetomidine has been found in 12 states, as well as Ontario and British Columbia, Canada and is most common where xylazine is common.^58,63^ For example, between March 2024 and March 2025, a study in Philadelphia found medetomidine in 83% of 260 fentanyl samples collected.^64^ Medetomidine is more potent and has a longer duration of action than xylazine, as it is more selective to α_2A_ receptors than xylazine.^63,65^ For this reason, medetomidine causes longer-lasting sedation than xylazine.^66,67^ Adding to its potential dangers, medetomidine is harder to detect than xylazine, since test strips have only recently become available for medetomidine, and it is often mixed with other opioid adulterants, including xylazine and tetracaine.^63,68^ This exposure to the sedatives xylazine and medetomidine together may have a synergistic effect, leading to even more severe respiratory depression, bradycardia, and reduced cardiac output.^57,63^

Although it is not entirely clear why medetomidine recently became popular as an opioid adulterant, researchers believe that it may be due to the crackdown on xylazine, which has caused the price of xylazine coming from China to double.^69,70^ At the same time, the crackdown on fentanyl in the United States seems to have led to the emergence of new highly potent synthetic opioids called nitazenes in Philadelphia, one of the cities where medetomidine use has been the most prevalent.^71^ It is also unclear if drug traffickers are illegally diverting medetomidine from veterinary supplies, or if they are manufacturing their own supply, either in the United States or in Mexico.^70^

### Pharmacology

Medetomidine is a racemic mixture of levomedetomidine **(6)** and dexmedetomidine **(7)**.^63^ Compared to clonidine **(3)**, medetomidine was found to be seven times more selective for α_2_ adrenergic receptors over α_1_ receptors.^72^ Medetomidine was found to have significantly higher affinity for α_2_ receptors than α_1A_ receptors in tests performed with human adrenergic receptors expressed in Chinese hamster ovary cells. Medetomidine had similar affinities for α_2A_ and α_2B_ receptors, with a significantly lower affinity for α_2C_ receptors.^73^

### Effects

The exact effect of medetomidine in humans is not well established.^71^ Similarly to other α_2_ agonists, medetomidine has been noted to cause bradycardia, hypotension, and sedation, as well as having analgesic and anxiolytic effects.^58,71^ However, it is clear that more research is needed on the effects of medetomidine on opioid users and its potential to increase the risk of opioid overdose. While no human studies have been performed on the potential for medetomidine withdrawal, it seems likely that this could occur, as both xylazine and dexmedetomidine can lead to withdrawal symptoms.^63,74^

### Toxicity

In the field of veterinary medicine, it is recommended that accidental exposure to medetomidine in humans be treated with the α_2_ antagonist atipamezole **(8)** at a dose of 25 mg administered every 2 min intramuscularly to a maximum dose of 5 mg of atipamezole per milligram of medetomidine exposure. However, it is noted that atipamezole may cause rebound sympathetic activation, including hypertension, and therefore is only safe for use in emergencies.^75^ Although the reversal of medetomidine with atipamezole has not been directly studied in humans, the reversal of dexmedetomidine with atipamezole was examined in a study of six (6) healthy male volunteers. Atipamezole was able to reverse the cardiovascular, sedative, and sympatholytic effects of dexmedetomidine at a dose of 60 parts atipamezole to one part dexmedetomidine, with both drugs showing an elimination half-life of about two (2) hours. The atipamezole had a rapid onset of action, and was well tolerated with the exception of one subject fainting and one experiencing bradycardia after atipamezole administration. However, administering atipamezole intravenously at a faster rate of 150 μg/kg in two (2) minutes led subjects’ norepinephrine levels to increase 10-fold.^76^ Overall, while atipamezole may be effective in reversing the effects of dexmedetomidine or medetomidine, its safety profile does not appear favorable for routine use.

### Pharmacokinetics

In addition to metabolism by CYP450 enzymes, medetomidine is metabolized by the UGT enzyme UGT2B10. This enzyme is found mainly in the liver, as well as in the kidney and intestine, and performs *N*_3_-glucuronidation **(9)** and some *N*_1_-glucuronidation **(10)** on medetomidine in the endoplasmic reticulum^77,78^ (Figure 4).

**Figure 4:**
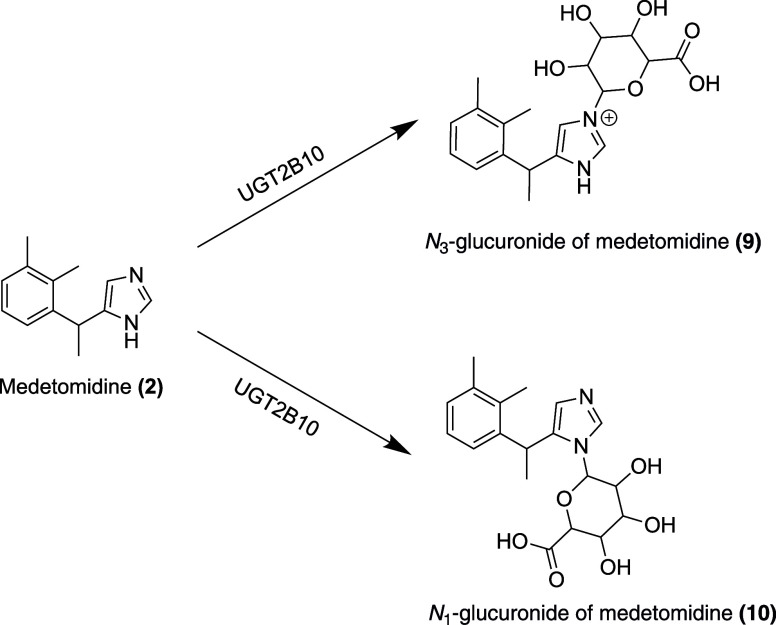
Metabolites of medetomidine (adapted from Kaivosaari et al.^78^).

In one of the few studies of medetomidine’s pharmacological effects in humans, healthy male volunteers tolerated doses of up to 120 μg intravenously without adverse effects, although decreased blood pressure and heart rate were recorded. The higher doses of 100 to 120 μg produced sedation, with an onset of 15 to 45 min and a duration of up to four (4) hours.^79^

### Physicochemical Description

Medetomidine is a crystalline solid. It is soluble in ethanol and DMSO.^80^ The p*K*_a_ of medetomidine is 7.1. Its log*D*_7.4_ is 2.89. Medetomidine is marketed as the HCl salt, which is a white to almost white powder that is freely soluble in water^81^ (Table 2).

### Structure–Activity Relationships

In a series of structure–activity relationship tests, the methyl group on the carbon bridge of medetomidine was found to be important for α_2_ receptor affinity, as removing this group decreased receptor affinity.^73^ Other small, nonpolar substituents did not significantly decrease receptor activity, but the methyl group gave the structure the greatest potency.^82^ It was also found to be essential that this group be lipophilic, as replacing the methyl group with a hydroxy or keto group decreased α_2_ receptor affinity. Furthermore, the conformational flexibility of this methyl group is needed in order for medetomidine to have α_1_/α_2_ receptor selectivity. In addition, the importance of stereochemistry was seen in that the *S*-isomer at the methyl position was a more potent agonist of α_2A_ and α_2C_ receptors than the *R*-isomer^73^ (Figure 5).

**Figure 5:**
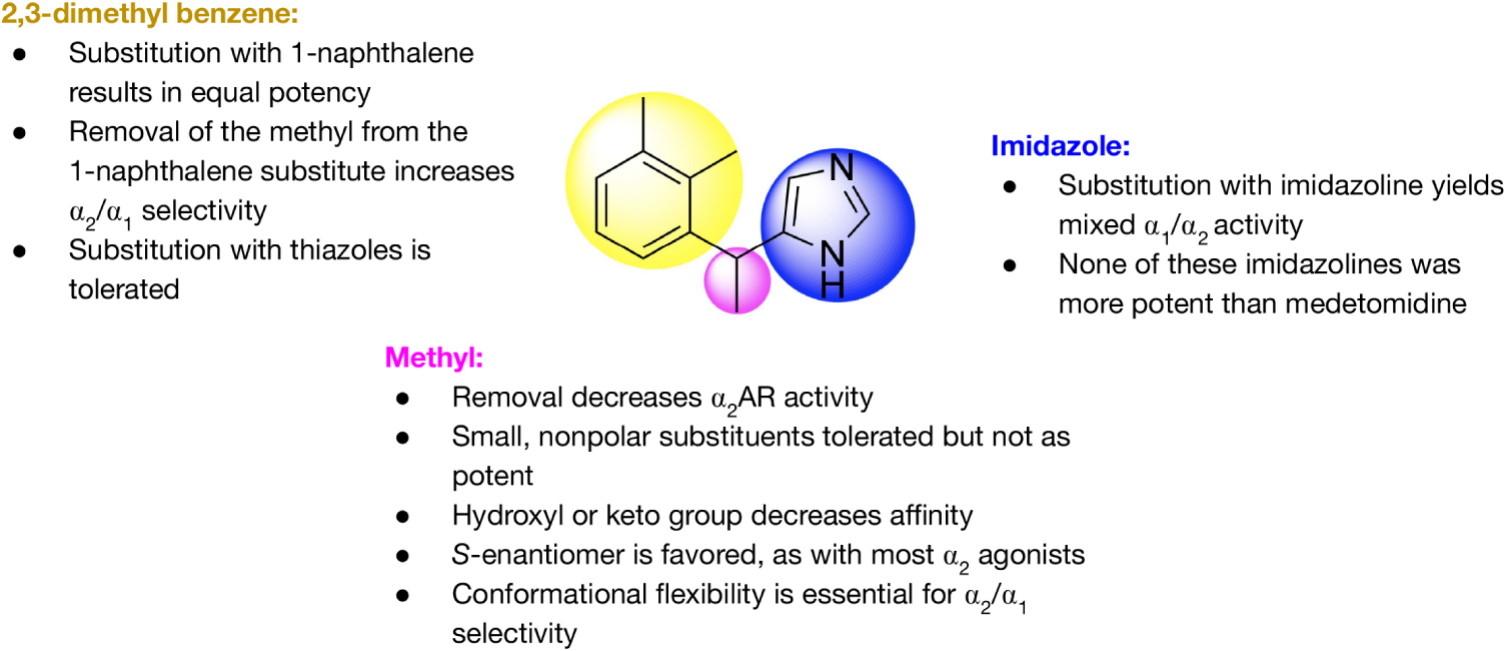
Structure–activity relationships of medetomidine **(2)** (adapted from de Andrade Horn et al.^5^ and Lalchandani et al.^73^).

### Other α_2_ Agonists Relevant to OUD

While α_2_ agonists other than xylazine **(1)** and medetomidine **(2)** have not yet been reported to be commonly used as opioid adulterants it seems probable that over time, as regulatory agencies crack down on known adulterants, demand will increase for other agents. In the following sections, we review other common α_2_ agonists and their pharmacology and chemistry. In addition, other α_2_ agonists have been found to be efficacious in reducing symptoms of withdrawal from opioids and facilitating abstinence and recovery from OUD.

## Dexmedetomidine

### Pharmacology

Dexmedetomidine **(7)** is approved by the FDA for sedation for surgical procedures, sedation of intubated patients in the ICU, and to treat agitation in patients with schizophrenia or bipolar disorder.^81,83^

Dexmedetomidine is the more pharmacologically active enantiomer of medetomidine. For example, dexmedetomidine is about 150-fold more potent than medetomidine at α_2C_ receptors^19,27^ (Table 1).

The α_2A_ receptor subtype is responsible for dexmedetomidine’s sedative, antinociceptive, and hypothermic effects.^84^ At high doses or when administered at a rapid rate, dexmedetomidine exhibits α_1_ as well as α_2_ activity.^83^ For example, in samples of rat aorta treated with the α_1_ agonist phenylephrine to induce contraction, high doses of dexmedetomidine attenuated this contraction in an apparent α_1_ blockade. This indicates that some of the dexmedetomidine may be able to occupy the α_1_ receptor without activating it, blocking phenylephrine from binding and acting as a competitive inhibitor.^85^ Side effects of dexmedetomidine may include somnolence and QT prolongation.^81^

Dexmedetomidine’s peripheral analgesic effects are mediated by G_i/o_ activation, which blocks the Na_v_1.8 sodium channels responsible for nociception, whereas G_s_ stimulation would lead to increased production of cAMP, thereby activating PKA to phosphorylate and activate Na_v_1.8. By coupling with G_i/o_, dexmedetomidine inhibits this effect, decreasing the excitability of dorsal root ganglion neurons.^86^

### Effects

Dexmedetomidine causes analgesia, sedation, decreased heart rate, and decreased oxygen consumption. It is also anxiolytic, making it beneficial in patients with significant preoperative stress. During surgery, dexmedetomidine improves hemodynamic stability and reduces shivering. Dexmedetomidine does not cause respiratory depression. Administration of dexmedetomidine postoperatively may be cardiac protective in patients receiving vascular surgery by reducing sympathetic outflow and thereby reducing myocardial oxygen demand.^87^

### Toxicity

Concurrent use of sublingual and intravenous dexmedetomidine may lead to withdrawal symptoms such as nausea and vomiting and agitation. Overdose of dexmedetomidine causes symptoms of bradycardia, hypotension, second-degree heart block, first-degree atrioventricular block, and cardiac arrest.^81^

### Pharmacokinetics

Dexmedetomidine is poorly absorbed orally, with a bioavailability of 16%, but when given sublingually or buccally, the bioavailability is 72% and 82%, respectively.^81,88^ Dexmedetomidine is rapidly distributed to a volume of distribution of 118 L.^83^ It is 94% protein bound.^83^ Like medetomidine **(2)**, dexmedetomidine is also metabolized by the UGT2B10 enzyme. However, while UGT2B10 is almost solely responsible for the metabolism of levomedetomidine **(8)**, UGT1A4 also plays a role in dexmedetomidine metabolism.^78^ UGT enzymes contribute more to the metabolism of levomedetomidine than to the metabolism of dexmedetomidine; more formation of the glucuronide metabolite was found for levomedetomidine than dexmedetomidine, and CYP450 enzymes were responsible for 3% of levomedetomidine clearance compared to 30% of dexmedetomidine clearance.^77^ Dexmedetomidine is also metabolized via aliphatic hydroxylation, mediated by CYP2A6, to form 3-hydroxy-dexmedetomidine **(11)**, and *N*-methylation to form 3-hydroxy *N*-methyl-dexmedetomidine **(12)** ^81,89^ (Figure 6). The primary mechanism of elimination is renal, with 95% of dexmedetomidine excreted in the urine.^83^ The terminal elimination half-life is approximately 2 h, and clearance is estimated to be approximately 39 L/hour^83^ (Table 3).

**Table 3:** Pharmacokinetic Parameters of Selected FDA-Approved α_2_ Agonists for Human Use^a^

	Dexmedetomidine	Clonidine	Lofexidine	Tizanidine	Guanfacine	Guanabenz
Onset of action	IV loading dose: 5–10 min.	Antihypertensive effect: Oral: IR: 0.5–1 h. (max. reduction in blood pressure: 2–4 h.);^90^ TD: Initial application: 2–3 days; steady state reached in ∼3 days.^111^		1–2 h.^142^		1 h.^170^
	Intranasal: 45–60 min.^195^					
Peak plasma effect	IV loading dose: 15–30 min.	IR: 1–3 h.;^90^ ER: 3–5 h.^197^	3–5 h.^120^	Fasting state: Capsule, tablet: 1 h.	IR: 2.6 h. (range: 1–4 h.)^153^	2–4 h.^170^
	IV continuous infusion: 60 min.^196^	Epidural: 19 ± 27 min.^91^		Fed state: Capsule: 3–4 h., Tablet: 1.5 h.^141^	ER: 4–8 h.^154^	
	Intranasal: 90–105 min.^195^	ER solution: 3–5 h.^92^				
Duration	Postcontinuous infusion (dose dependent): 60–240 min.^198–200^	Antihypertensive effect: 8–12+ hr.^104^		3–6 h.^142^	Antihypertensive effect: 24 h.^201^	Single dose: 6–8 h.;^170^ multiple dose: 10–12 h.^175^
Distribution	∼118 L; rapid^83^	∼2.9 L/kg;^111^ highly lipid soluble; distributes readily into extravascular sites^91^	Oral: 480 L; IV: 297.9 L^120^	IV: 2.4 L/kg^141^	IR: 6.3 L/kg^153^	7.4–13.4 L/kg^175^
Bioavailability	IM: 73% ± 11% (range: 54% to 91%)^202^	Oral: IR: 70–80%;^90^ ER: ∼89% (relative to IR formulation);^197^ ER solution: 96.6–97.1% (relative to ER tablets);^92^ TD: ∼60%.^111^	Oral: 72%^120^	∼40%;^141^ 21%^204^	IR: ∼80%^153^	75%^170^
	Intranasal: Median: 65% (range: 35% to 93%)^203^				ER (relative to IR): 58%^154^	
	Oral: 16% (range: 12% to 20%)^88^					
	SL: 72%					
	Buccal: 82%^81^					
Protein binding	94% ^81,83^	20–40%^91^	∼55%^120^	∼30%^141^	∼70%^153^	90%^175^
Elimination half-life (*t*_1/2_)	IV: 2 h.^83^	12–16 h.^90^	11–13 h. (first dose); 17–22 h. (steady state)^205^	∼2.5 h.^141^	IR: ∼17 h. (range: 10–30 h.)^153^	6 h.^170^
	SL: 2.8 h.^81^	Epidural administration: CSF *t*_1/2_: 1.3 ± 0.5 h.; plasma *t*_1/2_: 22 ± 15 h.^91^			ER: 18 ± 4 h.^154^	
		TD: (after patch removal): ∼20 h.^111^				
Excretion	Urine (95%); feces (4%)^81,83^	Oral: Urine (40–60% as unchanged drug)^90^	Urine (93.5%; 15–20% unchanged); feces (0.92%)^120^	Urine (60%); feces (20%)^141^	Urine (∼50% [40–75% of dose] as unchanged drug)^153^	Urine (<1% as unchanged drug)^175^
		Epidural: Urine (72%; 40–50% unchanged)^91^				
Clearance	39 L/h.^81,83^	Oral: Single dose: ∼0.25 L/kg/h.; Multiple dose: ∼0.4 L/kg/hour^206^		2 L/kg/h.^207^		11 L/kg/h.^208^

a IM: intramuscular; IR: immediate release; IV:intravenous; ER: extended release; TD: transdermal; SL: sublingual.

**Figure 6:**
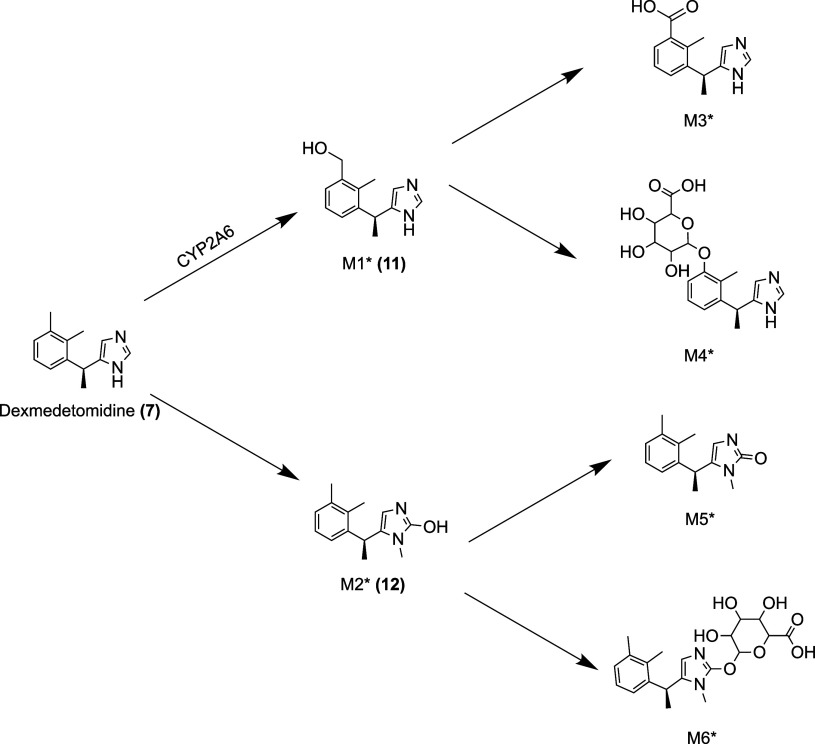
Metabolites of dexmedetomidine (adapted from Fernandes et al.^89^). M1*: 3-hydroxy-dexmedetomidine **(11)**. M2*: 3-hydroxy *N*-methyl-dexmedetomidine **(12)**. M3*: 3-carboxy-dexmedetomidine. M4*: *O*-glucuronide of 3-hydroxy-dexmedetomidine. M5*: 3-carboxy *N*-methyl-dexmedetomidine. M6*: dexmedetomidine-*N*-methyl *O*-glucuronide.

### Physicochemical Description

Dexmedetomidine is provided as an HCl salt, described by the chemical name 4-[(1*S*)-1-(2,3-dimethylphenyl)­ethyl]-1*H*-imidazole hydrochloride. Dexmedetomidine HCl is a white or off-white powder. It is freely soluble in water. Its p*K*_a_ is 7.1 and its log*D*_7.4_ is 2.89.^81^ Dexmedetomidine is available as a sublingual film or an intravenous solution (Table 2).

## Clonidine

### Pharmacology

Clonidine **(3)** is approved to treat hypertension;^90^ cancer pain, as an adjunct with opioids;^91^ and ADHD in children.^92^ Clonidine is also used off-label to treat opioid withdrawal,^93^ as well as vasomotor symptoms of menopause.^94^

Clonidine is a partial agonist at α_2_ receptors.^95^ Clonidine stimulates all three subtypes of the α_2_ receptor, α_2A_, α_2B_, and α_2C_, activating the G_i_ signaling pathway.^95,96^ This lack of selectivity may be responsible for clonidine’s large side effect profile.^96^ In hamster smooth muscle cells, clonidine’s effect as a partial agonist at α_2B_ receptors was demonstrated by the fact that it uncoupled G_i3_ from the α_2B_ receptor. As G_i3_ recruitment is necessary for an agonist to switch the receptor from G_i_ to G_s_ activity, clonidine prevents the G_i_ to G_s_ switch that full agonists, such as epinephrine, are able to effect.^26^ When used as sedatives, clonidine causes amnesia via coupling with G_i1_, G_i3_, and G_O1_.^97^

In addition to being an agonist at α_2_ receptors, clonidine is also a partial agonist at α_1A_ receptors, with an α_1A_ to α_2A_ selectivity ratio of only 1.2 ^98,99^ (Figure 2). Coadministration of clonidine and the α_1_ antagonist prazosin allowed clonidine to act as an effective analgesic in allodynic mice, showing that clonidine’s α_1_ activity diminishes its analgesic effects mediated by α_2A_ receptors.^99^

Clonidine is a mixed α_2_AR and imidazoline receptor (IR) agonist. Imidazolines that are more selective for IRs over α_2_ARs, such as rilmenidine **(13)** and moxonidine **(14)**, have demonstrated the existence of a distinct IR (Figure 7). These agents cause hypotension which is reversed by lower doses of idazoxan **(15)**, an IR antagonist, than the dose of idazoxan needed to reverse hypotension caused by a pure α_2_ agonist, such as α-methyldopa.^100^ While this phenomenon is less marked in clonidine, in another study, clonidine injected directly into the rostral ventrolateral medulla of rats caused a decrease in arterial pressure which was reversed by idazoxan but not by a selective α_2_ antagonist.^101^ Further research with highly selective IR ligands has shown that stimulation of the I_1_R is sufficient to elicit a hypotensive effect. It seems likely that the α_2_AR and IR work synergistically to produce hypotension, as low doses of a selective α_2_ agonist and a selective I_1_R agonist do not lead to hypotension when administered on their own, but do have a hypotensive effect when administered together.^102^ The mechanism behind the interaction between α_2_ARs and IRs is not yet fully understood; however, evidence has established that these two receptors can act on the same cAMP transduction pathway. In PC12 cells, which only express I_1_Rs and not α_2_ARs, benazoline **(16)**, a selective IR agonist, causes a decrease in cAMP levels after adenylyl cyclase stimulation via forskolin. Clonidine does not affect cAMP levels on its own, but it does antagonize the reduction in cAMP caused by benazoline. This shared pathway may only be one example of the interaction between adrenergic and imidazoline receptors, though, and further research will likely uncover additional aspects.^103^

**Figure 7:**
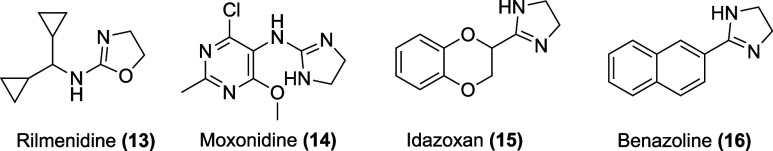
Structures of imidazoline receptor agonists and antagonists.

### Effects

Clonidine-induced stimulation of α_2_ receptors in the central nervous system reduces sympathetic outflow, resulting in vasodilation, decreased heart rate, and decreased renin secretion by the kidneys, ultimately reducing blood pressure.^90,104^ Clonidine also improves the sensitivity of the baroreceptor reflex, restoring the reduction of sympathetic tone that occurs in response to elevated blood pressure in patients with hypertension.^104^

### Toxicity

Clonidine’s side effects include hypotension, dizziness, dry mouth, drowsiness, sedation, and constipation.^90,91^ Activation of the α_2A_ receptor subtype is responsible for clonidine and dexmedetomidine’s sedative and analgesic effects, as well as its ability to reduce the amount of anesthetic drugs needed to sedate patients.^105^

Abrupt cessation of clonidine therapy may result in withdrawal symptoms of agitation, restlessness, headache, and rebound hypertension, as well as a rebound rise in catecholamine levels. Overdose with clonidine leads to symptoms such as hypotension, bradycardia, respiratory depression, weakness, drowsiness, and hypothermia.^90^ There is not a clearly defined toxic dose for clonidine, with some sources reporting cardiovascular and respiratory depression after a dose of 0.01 to 0.02 mg/kg and other sources reporting effects after a dose of 0.2 mg.^106^

Although clonidine has been used in the treatment of opioid withdrawal, it may be abused and become addictive. Among PWUD, it is used for its calming effect and to enhance the effects of opioids, like methadone, heroin, or painkillers. Opioid users tend to start using clonidine when the drug is provided to them in a rehabilitation program to help manage withdrawal symptoms, and users then find that clonidine is inexpensive and readily available, as physicians are not always aware of its potential for abuse.^107,108^ Clonidine use tends to present similarly to intoxication, with signs including drowsiness, unstable mood, low blood pressure, and depressive symptoms. With chronic use, abrupt cessation of clonidine leads to withdrawal symptoms such as tremors, nausea, tachycardia, and hypertension. The fact that these symptoms are rapidly alleviated by taking more clonidine leads to a reinforcing addiction-like pattern in individuals who are already prone to addictive behavior. By causing further withdrawal symptoms itself, clonidine use may cause addicts to increase their clonidine dose or ask for their methadone dose to be increased.^109^ A survey of 48 applicants to methadone maintenance programs found that 46% had ever used clonidine, 18% currently used clonidine, and 35% thought it was addictive. Similarly, among 30 methadone patients who had told a counselor they were using clonidine, the survey results showed that 63% were current users, 37% reported craving clonidine, and 90% thought it was addictive.^107^ A case report of a 66-year-old male with a history of substance abuse noted that the patient frequently went to the emergency room to try to obtain clonidine, took the drug in large quantities, and had symptoms of withdrawal if he ceased taking clonidine.^108^ In the locus coeruleus, opiate withdrawal is linked to an increased firing rate of noradrenergic neurons. A study in morphine-dependent rats found that clonidine reduced this firing rate, thereby ameliorating withdrawal symptoms.^110^

### Pharmacokinetics

Clonidine has an absolute bioavailability of 70 to 80% when given orally in the immediate-release formulation.^90^ It is highly lipophilic, tending to distribute into the extravascular space, with a volume of distribution of about 2.9 L/kg.^91,111^ The enzyme CYP2D6 hydroxylates clonidine to form the metabolite 4-hydroxyclonidine **(17)** ^112^ (Figure 8). Clonidine undergoes enterohepatic circulation.^113^ Clonidine is excreted renally, with 40 to 60% of the dose excreted as unchanged drug.^90^ Clonidine has a half-life of 12 to 16 h when given intravenously^90^ (Table 3).

**Figure 8:**
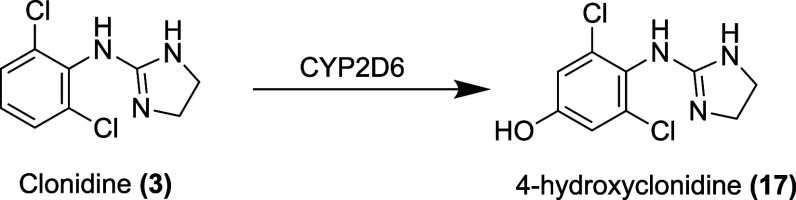
Metabolites of clonidine.

### Physicochemical Description

Clonidine is provided as an HCl salt described by the chemical name 2-(2,6-dichlorophenylamino)-2-imidazoline hydrochloride. Clonidine HCl is a white crystalline solid. It is soluble in water and alcohol.^90^ Clonidine is available as an oral tablet, a once-weekly transdermal patch, an epidural solution, or an extended-release solution (Table 2).

### Structure–Activity Relationships

QSAR studies have demonstrated that steric hindrance at the ortho position, occupied by the bulky chlorine moiety in the structure of clonidine, is highly important in determining the action of α-adrenergic agonists.^114,115^ Clonidine’s other ortho substituent, another chlorine, also contributes to its antihypertensive activity by minimizing resonance between the aromatic ring and the imidazolidine ring. The lack of a para substituent also contributes to clonidine’s mechanism of action, although it is not as critical; compounds with small para substituents such as a hydroxyl group had similar antihypertensive activity in rats. Lipophilicity alone is not sufficient to predict hypotensive efficacy, although this factor is key in the pharmacokinetics of an antihypertensive. Compounds similar to clonidine that are too lipophilic penetrate the blood–brain barrier quickly and rapidly achieve a maximum decrease in blood pressure, but they also recross the blood–brain barrier quickly, leading to a short duration of action. In contrast, the less lipophilic clonidine takes longer to reach the maximal decrease in blood pressure, but has a longer-lasting hypotensive effect because it stays in the brain longer.^116^

### Applications in OUD

While clonidine misuse by opioid users can augment patterns of dependence and addictive behavior, like other α_2_ agonists, clonidine also shows promise as a treatment to ameliorate symptoms of opioid withdrawal. In a study of 127 opioid-dependent individuals receiving inpatient treatment, subjects were randomized to receive very-low dose naltrexone, clonidine, or very-low dose naltrexone and clonidine. The naltrexone and clonidine group showed better attenuation of withdrawal symptoms than the naltrexone-only or clonidine-only groups.^93^ While this result could be explained by the fact that clonidine and naltrexone have different mechanisms of action and can therefore attenuate withdrawal symptoms via different pathways, several studies have shown that there is more to this synergistic relationship. When morphine binds and activates the mu opioid receptor, it forms a heterodimer with the α_2A_ adrenergic receptor and inactivates it via a conformational change.^117^ Because of the cross-talk between these receptors, naltrexone not only blocks mu opioid receptors, but in doing so, it also reduces the norepinephrine efflux that is a hallmark of opioid withdrawal.^118^ Concurrent treatment with the α_2A_ agonist clonidine then further decreases excess norepinephrine release and better manages withdrawal symptoms. This synergistic effect means that combining clonidine with naltrexone instead of using clonidine alone would allow for a lower dose of clonidine to be used, thereby minimizing clonidine’s adverse effects, while still allowing patients to benefit from better control of withdrawal symptoms than that which can be obtained with an opioid antagonist alone.^93^ In addition, clonidine’s IR activity may play a role in its beneficial effects in the treatment of OUD. In a study performed in rats, both clonidine and the IR-selective agent **14** reduced cue-induced reinstatement of cocaine-seeking behavior, suggesting a further role for mixed α_2_AR and I_1_R agonists in the development of therapeutic agents for the treatment of OUD.^119^

## Lofexidine

### Pharmacology

Lofexidine **(5)** was approved by the FDA in 2018 to treat opioid withdrawal. It is typically used in the first 5 to 7 days of discontinuing opioid use to address the acute symptoms of withdrawal.^120^

In addition to being an agonist at α_2_ adrenergic receptors, lofexidine is an agonist at serotonin 5-HT_1_ receptors (Figures 9 and 2). This gives lofexidine anxiolytic and antidepressant properties.^121^ In a study of mice treated with naloxone, the α_2A/2C_ agonist cyclomethyline **(18)** reduced withdrawal symptoms, and had an antidepressant effect via the activation of 5-HT_1A_ receptors.^122^ Allyphenyline **(19)**, which has a similar structure to **18**, prevented mice from developing morphine tolerance or dependence.^123^ Allyphenyline was also found to be effective in treating alcohol withdrawal in rats.^124^ A structure–activity relationship study of **19** at human 5-HT_1A_ receptors found that the structure of the carbon bridge was essential for 5-HT_1A_ agonism. Polar moieties, such as oxygen- or nitrogen-containing groups, were needed for the molecule to show affinity for the 5-HT_1A_ receptor. Methyl groups on the carbon bridge also increased affinity. Regarding the benzene ring, the presence of an ortho substituent led to better affinity than a para or meta substituent, with higher steric hindrance at the ortho position resulting in greater affinity^125^ (Figure 10). Biphenylene **(20)**, another α_2_ and 5-HT_1A_ agonist with a similar structure to **19**, was used to treat rats with neuropathic pain and was found to cause a decrease in pain and hypersensitivity.^126^ In all, these results suggest an additional mechanism of action for lofexidine wherein its antidepressant and antinociceptive properties increase its efficacy as an agent for opioid detoxification.

**Figure 9:**
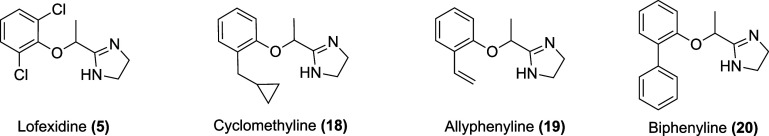
α_2_ and 5-HT_1A_ dual agonists.

**Figure 10:**
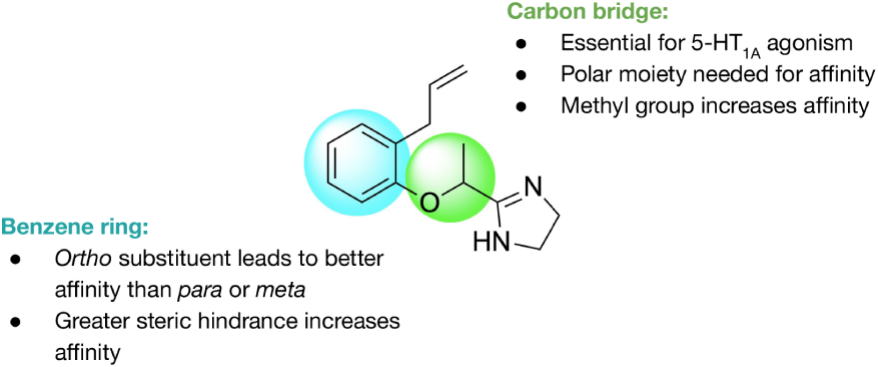
Structure–activity relationships of allyphenyline **(19)** at 5-HT_1A_ receptors (adapted from Del Bello et al. 2016 ^125^).

### Toxicity

Side effects of lofexidine include hypotension, bradycardia, syncope, and QT prolongation. Abrupt cessation of treatment may cause rebound hypertension.^120^ Because lofexidine has higher specificity to α_2A_ receptors than clonidine, lofexidine is less likely to cause hypotension as a side effect than clonidine.^127–130^ For example, in a study of 80 patients comparing lofexidine and clonidine in the treatment of heroin withdrawal, treatment had to be withheld because of hypotension on 19 of 188 total treatment days (10.1%) for lofexidine versus 34 of 162 treatment days (20.9%) for clonidine.^131^ Another study of 28 patients receiving treatment for opiate addiction recorded fewer incidences of hypotension (53% vs 93%; data not significant) and adverse drug events (114 vs 226) for lofexidine compared to clonidine, as well as less patients reporting feeling unwell (14% vs 86%) or lethargic (21% vs 43%).^132^ Overdose of lofexidine may lead to symptoms of hypotension, bradycardia, and sedation.^120^

### Pharmacokinetics

Lofexidine has an absolute oral bioavailability of 72%, and is widely distributed, with a volume of distribution of 480 L.^120^ Lofexidine is metabolized via *O*-dealkylation, yielding 2,6-dichlorophenol **(21)**, which is then excreted in the form of two *O*-glucuronic acid conjugates, which are biologically inactive^133^ (Figure 11). Lofexidine is primarily excreted in the urine, with 15 to 20% of the dose recovered as unchanged drug.^120^ Its half-life is 17 to 22 h at steady state^120^ (Table 3).

**Figure 11:**
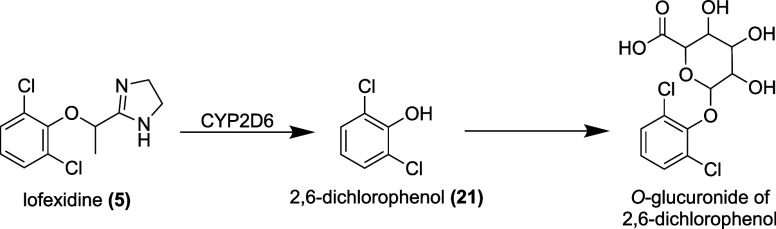
Metabolites of lofexidine.

### Physicochemical Description

Lofexidine HCl (2-[1-(2,6-dichlorophenoxy)­ethyl]-4,5-dihydro-1*H*-imidazole monohydrochloride) is a white or off-white crystalline powder. It is freely soluble in water, methanol, and ethanol, while in chloroform, it is slightly soluble, and in *n*-hexane and benzene, it is practically insoluble^120^ (Table 2).

### Applications in OUD

Lofexidine has been shown to be effective at reducing withdrawal symptoms in opioid users. A study comparing lofexidine to a placebo among opioid users with withdrawal symptoms over 8 days found that by day three, scores on the Subjective Opioid Withdrawal Symptoms (SOWS) questionnaire were lower for the lofexidine group than the placebo group. Patients in the lofexidine group were significantly more likely to experience hypotension, dizziness, and bradycardia than patients receiving the placebo.^134^ In a similar study with 602 patients, patients receiving lofexidine had fewer withdrawal symptoms and a higher study completion rate than patients receiving the placebo over seven (7) days.^135^

Lofexidine has generally been found to be more effective than clonidine at managing opioid withdrawal symptoms. A systematic review of five studies comparing lofexidine and clonidine found that only one study reported lofexidine to be significantly better than clonidine at managing withdrawal symptoms,^136^ while the other four studies found no significant difference.^137^ However, a retrospective chart review of 166 patients who received lofexidine for outpatient opioid withdrawal treatment and 432 patients who received clonidine found that 63% of patients treated with lofexidine achieved opioid-free status after 30 days, compared to 21% of patients receiving clonidine. Lofexidine also showed higher rates of opioid-free status at six months. The authors note that lofexidine more effectively managed patients’ withdrawal symptoms and allowed patients to transition to long-term recovery plans, such as MOUD.^138^

A study of 57 patients who had gone through detoxification for OUD compared the efficacy of lofexidine plus naltrexone and placebo plus naltrexone for ameliorating withdrawal symptoms and supporting abstinence from opioids. Although there were no significant differences in patients’ subjective opioid withdrawal symptoms, the lofexidine group showed a significant improvement in control over opioid craving from baseline to week 12, while the placebo group did not. In a secondary posthoc analysis of only patients who completed the study, more patients from the lofexidine group were opioid-free, and patients in this group reported better naltrexone adherence and fewer opioid cravings compared to the placebo group. These results suggest that by helping to decrease cravings, lofexidine in combination with an opioid agonist may increase patients’ chances of completing treatment and avoiding relapsing into opioid use.^139^ A similar study of 18 patients also showed reduced relapse rates and higher opioid abstinence rates in patients receiving lofexidine and naltrexone over placebo plus naltrexone. The researchers also used imagery exposure to measure the effects of stress on opioid cravings. Patients in the lofexidine-naltrexone group had on average lower heart rates following the induced stress and fewer resulting opioid cravings than the placebo-naltrexone group. Opioid withdrawal is associated with noradrenergic hyperactivity, and while this effect usually diminishes during abstinence, the authors suggest that noradrenergic hypersensitivity may persist, allowing stress to induce opioid cravings. As an α_2_ agonist, lofexidine decreases noradrenergic activity, thereby reducing stress-related cravings.^140^

## Tizanidine

### Pharmacology

Tizanidine **(22)** is FDA-approved to treat muscle spasm and associated pain.^141^ It is also used off-label for spasticity associated with cerebral palsy, as an adjunct in headache treatment, and for lower back pain.

Similarly to clonidine, tizanidine is an agonist at α_1_ receptors, with an α_1A_ to α_2A_ selectivity ratio of 1.0, reflecting its equal potency at the two receptors (Figure 2). Coadministration of an α_1A_ antagonist in rats increased the potency of tizanidine as an analgesic without affecting the dose needed to cause sedation, effectively increasing the therapeutic window between tizanidine’s analgesic and sedative effects, which usually occur at roughly the same dose.^99^

Tizanidine binds to the α_2_AR with high affinity and is highly potent. Tizanidine binds to α_2A_ receptors with over 3,000 times more affinity than clonidine^19,27^ (Table 1).

### Effects

Tizanidine decreases the excitability of spinal motor neurons by increasing the presynaptic inhibition of motor neurons. This effect is most prominent at polysynaptic pathways, due to the increased number of connections between the spinal and motor neurons. This effect causes a decrease in muscle spasticity by inhibiting neuronal firing.^142^

### Toxicity

Side effects of tizanidine include sedation, hallucinations, and hypotension, especially in patients taking antihypertensives.^141^ Tizanidine is noted to have fewer cardiovascular side effects than clonidine.^128^

Tizanidine use can lead to dependence and withdrawal, especially in patients who use high doses, receive tizanidine for a long duration, or are concomitantly receiving opioids. For this reason, it is recommended that tizanidine dosing be tapered when discontinuing therapy. Withdrawal symptoms include hypertension, hypertonia, tachycardia, anxiety, and tremor.^141^ These symptoms are due to the fact that α_2_ agonists inhibit norepinephrine release, and cessation of this chronic blockade leads to a surge in plasma norepinephrine levels.^143^ A similar sympathetic surge is seen in clonidine withdrawal.^144,145^ Tizanidine withdrawal has been treated with benzodiazepines or dexmedetomidine, in concert with a tapering of the tizanidine dose, in various case reports.^146,147^ Overdose with tizanidine is characterized by somnolence, lethargy, confusion, and coma, as well as hypotension and bradycardia.^141^

### Pharmacokinetics

The absorption of tizanidine is affected by food. Compared to the fasting state, administration of tizanidine tablets in the fed state led to a 30% higher *C*_max_, 30% greater total absorption, and an increase in *T*_max_ from 1 to 1 h and 25 min. For this reason, it is recommended that patients consistently take tizanidine always in the fed state or always in the fasting state.^141^ Tizanidine has a volume of distribution of 2.4 L/kg when given intravenously.^141^ Tizanidine undergoes extensive first-pass metabolism in the liver, wherein CYP1A2 degrades the drug to form two primary inactive metabolites, (5-chloro-4-(2-imidazolin-4-on-2-ylamino)-2,1,3-benzothiazdiazole **(23)** and 5-chloro-4-(guanidino)-2,1,3-benzothiadiazole **(24)** ^141,148,149^ (Figure 12). Sixty percent (60%) of the dose of tizanidine is excreted in the urine, and 20% is excreted in the feces.^141^ Tizanidine has a half-life of 2.5 h^141^ (Table 3).

**Figure 12:**
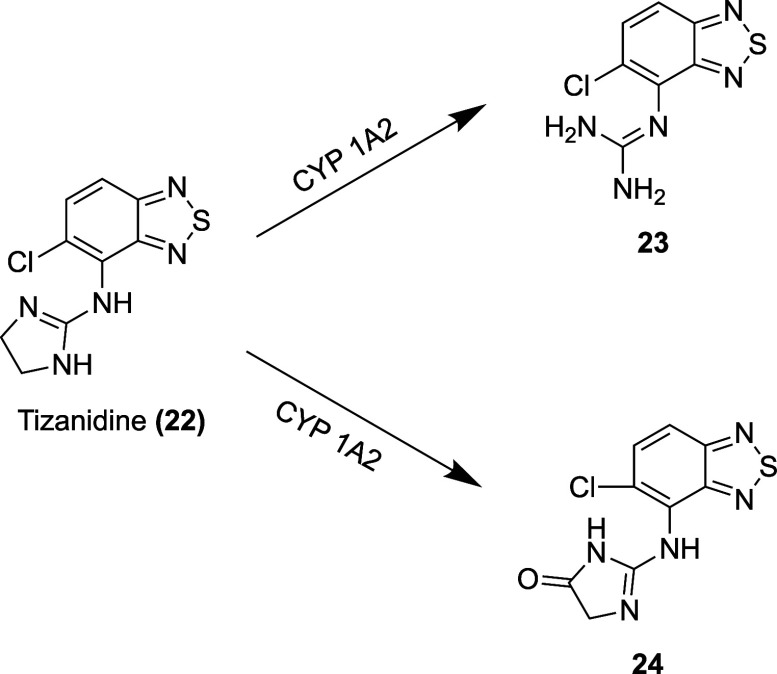
Metabolites of tizanidine.

### Physicochemical Description

Tizanidine is prepared as an HCl salt, with the chemical name 5-chloro-4-(2-imidazolin-2-ylamino)-2,1,3-benzothiadiazole monohydrochloride. Tizanidine HCl is a crystalline powder that is white or off-white in color. It is slightly soluble in methanol and water, and its solubility increases with decreasing pH^141^ (Table 2).

### Applications in OUD

A study of the metabolism of opioids in human liver microsomes found that tizanidine strongly inhibits the CYP2D6-mediated metabolism of the opioid oxycodone. While this is only one pathway affecting one drug, the authors note that skeletal muscle relaxants such as tizanidine may decrease the efficacy of opioids or lead to toxicity due to a lack of metabolic breakdown.^150^

Rats treated with either morphine, naloxone, or morphine and naloxone were administered tizanidine or clonidine to control withdrawal symptoms. Tizanidine was more effective than clonidine at controlling withdrawal symptoms of increased salivation, increased urine and feces output, and wet dog shaking. In the morphine group, tizanidine treatment led to hypothermia, while in both groups, tizanidine treatment led to increased pain threshold.^151^ In a study of 16 (16) heroin addicts treated with tizanidine and ten heroin addicts in a control group, all patients were retained through the 10-day acute withdrawal period, but treatment with tizanidine significantly reduced the severity of all seven (7) types of withdrawal symptoms analyzed.^152^

## Guanfacine

### Pharmacology

Guanfacine **(25)** is approved by the FDA to treat hypertension^153^ and to treat ADHD in children.^154^

Guanfacine is selective for α_2A_ receptors, and among the α_2_ agonists, it is the most selective known α_2A_ agonist.^155^ Similarly to clonidine, guanfacine can act as an agonist at postsynaptic α_1_ receptors.^98^

### Effects

In children with ADHD, acute treatment with immediate-release guanfacine leads to a decrease in heart rate, systolic blood pressure, and diastolic blood pressure, while these values return to baseline with chronic use.^156^ In treatment with extended-release guanfacine, a long-term, modest decrease in systolic and diastolic blood pressure. Guanfacine has not been shown to prolong the QT interval.^157^ In patients with hypertension, once-daily guanfacine lowers blood pressure, as well as decreasing heart rate, but not to a clinically significant degree.^158^

### Toxicity

Side effects of guanfacine may include hypotension, bradycardia, syncope, somnolence, and sedation. Rebound hypertension has been observed upon cessation of treatment.^154^

Symptoms of overdose with guanfacine include hypotension, bradycardia, drowsiness, and lethargy.^153^

### Pharmacokinetics

Guanfacine is well absorbed orally, with a bioavailability of 80%. It has a volume of distribution of 6.3 L/kg.^153^ The metabolism of guanfacine is primarily carried out by CYP3A4, generating 3-hydroxy-guanfacine **(26)** (Figure 13). Hepatic metabolism accounts for about 50% of guanfacine clearance.^154,159^ About 50% of guanfacine is excreted in the urine as unchanged drug.^153^ Immediate-release guanfacine has a half-life of 17 h^153^ (Table 3).

**Figure 13:**
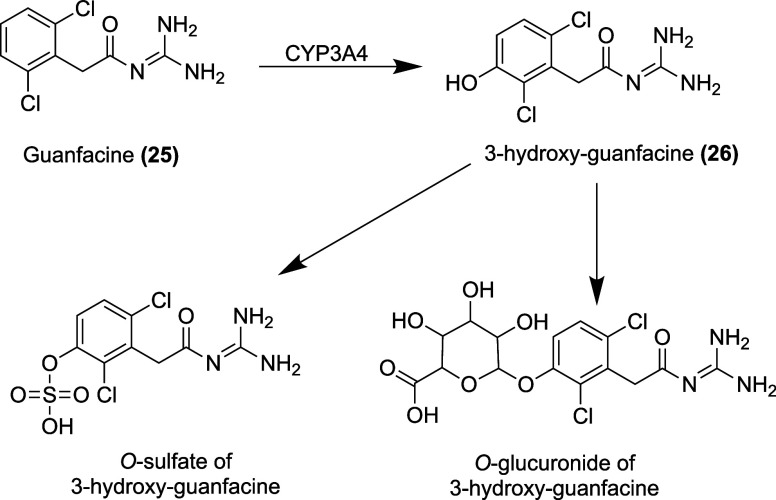
Metabolites of guanfacine.

### Physicochemical Description

Guanfacine is provided as an HCl salt, described by the chemical name *N*-amidino-2-(2,6-dichlorophenyl)­acetamide monohydrochloride. Guanfacine HCl is a white or off-white crystalline powder. It is slightly soluble in acetone and sparingly soluble in alcohol and water. In methanol, guanfacine is much more soluble, with a solubility of over 30 mg/mL^154^ (Table 2).

### Applications in OUD

In a six-month study of 75 patients receiving guanfacine and naltrexone and 75 patients receiving naltrexone only, the addition of guanfacine to MOUD treatment did not lead to better treatment retention or more patients reaching opioid-free status, but it did reduce stress, as measured by the Perceived Stress Scale, and reduced cravings for opioids. Patients in the guanfacine group reported mild side effects, including headache, anorexia, insomnia, and dizziness.^160^ A study of guanfacine in smoking cessation found similar results to those seen with lofexidine for opioid withdrawal: stress increased cravings to smoke and shortened time to resist smoking in the placebo group, but not in the guanfacine group. Like lofexidine, guanfacine diminishes the stress response by reducing norepinephrine and dopamine release.^161^

Guanfacine also strengthens network connections in the prefrontal cortex, preventing stress from impairing prefrontal cortex function and allowing for better self-control. The authors note that guanfacine significantly reduced systolic blood pressure, as well as decreasing diastolic pressure and heart rate, and therefore may not be suitable for patients with hypotension.^161^ Furthermore, in rats with induced morphine withdrawal, guanfacine reversed the accelerated norepinephrine turnover in the cerebral cortex associated with withdrawal, although clonidine achieved the same effect with much higher potency.^162^ Guanfacine appears to have a sex-dependent mechanism of action. In a study of 13 female and 27 male cocaine-dependent individuals presented with stress and drug cue scripts, treatment with guanfacine for three (3) weeks attenuated cocaine and alcohol cravings, anxiety, and anger and sadness compared to placebo in women, but not in men, while reducing nicotine cravings in both groups.^163^

Guanfacine has also been shown to enhance cognitive performance, as it binds postsynaptic α_2A_ receptors in the prefrontal cortex as an agonist to the endogenous ligand norepinephrine, thereby stimulating the same responses of increased attention and arousal.^164^ Further research in rats suggests that stimulation of α_2A_ receptors in the prefrontal cortex also suppresses postsynaptic glutamate release, leading to the hypothesis that guanfacine may balance excitatory inputs by both enhancing excitatory inputs, via the G_i_–cAMP–HCN pathway, and inhibiting excitatory inputs, via the G_i_–cAMP–PKA–PP1 pathway. This balance mechanism would allow guanfacine to maintain optimal levels of arousal in stressful situations.^165^ Through a feedback inhibition mechanism, guanfacine also decreases the synthesis of norepinephrine in the brain, further mediating an appropriate balance of excitatory signaling.^166^ Guanfacine facilitates working memory by enhancing delay-related firing in neurons in the prefrontal cortex to increase connectivity.^167,168^ This is the same mechanism of action by which guanfacine treats ADHD. By stimulating α_2A_ receptors in the prefrontal cortex to release norepinephrine, guanfacine improves cognitive performance and working memory.^169^ Together, these effects suggest that guanfacine works through a variety of mechanisms to address both symptoms of withdrawal and the chemical pathways potentiating addictive behavior.

## Guanabenz

### Pharmacology

Guanabenz **(27)** is used to treat hypertension.^170^ While guanabenz is highly selective toward α_2A_ receptors, it also acts as an antagonist at α_1_ receptors.^98^ Like clonidine, guanabenz causes amnesia via coupling with G_i1_, G_i3_, and G_o1_.^97^

### Effects

Guanabenz has additional effects on the central nervous system independent of its α_2_ adrenergic agonist activity. In mice genetically modified to model Parkinson’s disease, guanabenz prevented 6-hydroxydopamine from inducing cell death and inhibited the loss of parkin, which has a protective effect on neurons.^171^ In a rat model of Alzheimer’s disease, guanabenz attenuated the phosphorylation of tau proteins, inhibited the increase in amyloid precursor proteins, lowered levels of reactive oxygen species, and improved memory impairment by lowering the escape latency time in a rat maze. While the mechanism underlying this effect is not yet known, it may involve guanabenz’s ability to reduce endoplasmic reticulum stress by neutralizing reactive oxygen species and blocking apoptotic factors and caspases.^172^

Alpha-2 agonists have been shown to bind to α_2_ receptors on subcutaneous fat cells, leading to inhibited lipolysis. Administration of 4-hydroxy-guanabenz, the main metabolite of guanabenz and a partial agonist at α_2_ receptors, led to a decrease in body weight and lower blood glucose, triglycerides, and cholesterol in male obese rats.^173^ Guanabenz itself decreased gastric emptying in rats and induced a reduction in caloric intake when given over a course of 25 days.^174^

### Toxicity

Guanabenz often causes sedation.^170^ Limited data are available on accidental overdose with guanabenz, but in two pediatric patients, symptoms of overdose included hypotension, bradycardia, irritability, miosis, somnolence, and lethargy.^170^

### Pharmacokinetics

Guanabenz has an oral bioavailability of 75%.^170^ Its volume of distribution is 7.4 to 13.4 L/kg, and it is 90% protein bound.^175^ The main metabolite of guanabenz is 4-hydroxy-guanabenz **(28)**, which is a partial agonist at α_2A_ receptors.^173^ Another metabolite, guanoxabenz **(29)**, is formed via *N*-hydroxylation mediated by CYP1A2 enzymes in the liver. In certain liver samples taken from human subjects, a bioreversible, cyclical reaction was observed where guanoxabenz was returned to guanabenz via *N*-dehydroxylation.^176^ This reverse reaction was catalyzed by an enzyme system made of cytochrome *b*_5_, NADH cytochrome *b*_5_-reductase, and benzamidoxime reductase^176,177^ (Figure 14). This enzyme system is found in mitochondria in the liver and kidneys, as well as in microsomes of various organs, including the liver, kidneys, brain, intestine, and lungs.^178^ Results of the Ames test for guanoxabenz showed toxicity to two strains of *S. typhimurium*; however, since the conversion of guanoxabenz to guanabenz is more efficient than the conversion of guanabenz to guanoxabenz, the toxicity of guanoxabenz is likely of little concern.^179^ While accounting for much less of the clearance of guanabenz, another main metabolite is 2,6-dichlorobenzaldehyde azine and its *N*-glucuronide, both of which are inactive.^175^ Guanabenz is mainly eliminated in the urine, with only 1% of the dose remaining as unchanged drug.^174,175^ Guanabenz has a half-life of 6 h^170^ (Table 3).

**Figure 14:**
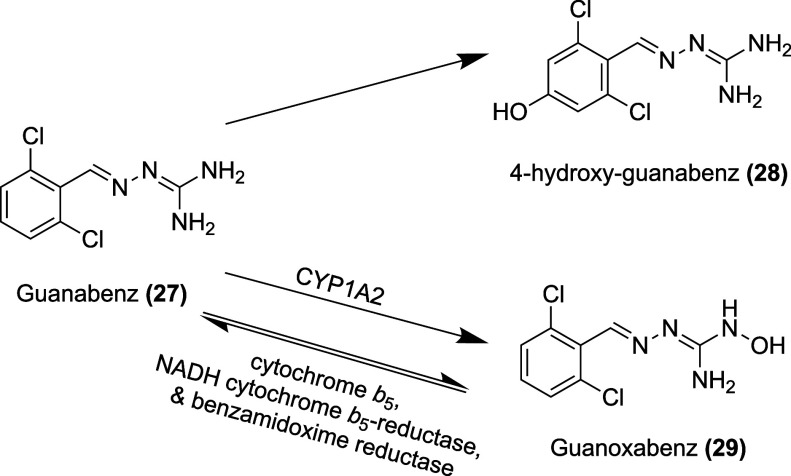
Metabolites of guanabenz.

### Physicochemical Description

Guanabenz is provided as an acetate salt with the chemical name 2,6-dichlorobenzylideneaminoguanidine acetate. Guanabenz acetate is a white or off-white crystalline powder. It is soluble in alcohol and sparingly soluble in water^170^ (Table 2).

### Applications in OUD

While limited studies have been performed on the potential of guanabenz as a treatment for opioid withdrawal, one study examined the efficacy of guanabenz among a group of 47 opioid users. Although only five (5) patients were retained for the entire 14 days of the study, with a mean retention of five (5) days, 70.2% of the subjects reported that guanabenz suppressed withdrawal symptoms. No patients showed a reduction in systolic or diastolic blood pressure of more than 10 mmHg during the course of the study.^180^ A case report of a 29-year-old male entering inpatient treatment for codeine addiction notes that the patient had severe side effects from clonidine, including agitation and tachycardia, and was therefore switched to treatment with guanabenz on day 6 of treatment. The patient received 4 mg of guanabenz every eight (8) hours, with the dose tapered on days 13 through 15 of treatment, and all withdrawal symptoms improved.^181^

## Conclusions

The α_2_ agonists reviewed in this paper have little structural diversity, all sharing a benzene-imidazole scaffold that is able to activate the α_2_AR (Figure 1). While they have diverse pharmacokinetic properties, this structural similarity means that any of these agents could be the next major adulterant of fentanyl, with unknown consequences. Given that xylazine is many times less potent than other α_2_ agonists (Figure 2), it seems plausible that should other α_2_ agonists be used as adulterants of fentanyl, the consequences may be even more detrimental than the effects of fentanyl adulteration with xylazine. Many are readily available due to their licit uses in a variety of disease states, and their simple structures mean that they could easily be synthesized. Xylazine and medetomidine have already been found as adulterants of the opioid supply, while clonidine is known to be abused either to ameliorate withdrawal symptoms or to enhance the effects of opioids. In contrast, clonidine, lofexidine, tizanidine, and guanabenz have shown efficacy when used to treat OUD to alleviate symptoms of withdrawal and promote abstinence. Guanfacine has also been shown to reduce stress and improve cognition, indirectly supporting addiction recovery. Rigorous monitoring of the presence of any of these agents in the illicit drug supply is crucial in order to best predict trends in α_2_ agonist use and quickly mobilize responses to minimize the health consequences for PWUD. At the same time, further research into the efficacy of α_2_ agonists in the treatment of OUD may lead to more effective management of withdrawal symptoms, giving PWUD a better chance of successfully becoming and remaining abstinent. A focus on developing new agents for the treatment of OUD that contain distinct structural features which prevent their abuse or use as adulterants would further take advantage of their potential while minimizing the possibility for harm.

The most pressing need in combating the spread of α_2_ agonist-adulterated fentanyl is for an antidote that would reverse the effects of α_2_ agonist exposure, including bradycardia and respiratory depression. In the absence of such an agent, other public health measures are needed to better serve PWUD. One simple intervention would be to make xylazine test strips freely and readily available so that PWUD can know if drugs they are considering taking are adulterated with xylazine. However, in areas where xylazine has become ubiquitous in the drug supply, test strips may not be as beneficial, especially as many individuals in these areas may be actively seeking out xylazine due to withdrawals.^182^ On the other hand, spreading awareness of the risks of xylazine in areas where it is less common may prove efficacious. Increased understanding and training among law enforcement and emergency medical personnel is crucial, as these individuals are on the front line and may play the deciding role in determining if a patient receives care. In all, to truly ameliorate this growing crisis, holistic interventions targeting PWUD, first responders, and hospital personnel are needed that address the need for wound care and addiction recovery services while staying abreast of the ever-changing illicit drug market.

We propose a concerted effort to develop an α_2_ agonist reversing agent that is an antagonist at α_2_ARs. A biased or functionally selective ligand may be useful in order to further develop the specificity of the reversal agent to counter the most life-threatening effects of an α_2_ agonist overdose, namely respiratory depression and bradycardia, without producing any rebound effects. As there are no existing FDA-approved reversal agents available, there is a critical and urgent need for research in this area.

Methods. We researched through the National Library of Medicine, Google Scholar and Patents, using keywords that included: Opioid Use Disorder, OUD, adrenergic, alpha-2 adrenergic agonist, xylazine, medetomidine, clonidine, public health, opioid overdose, α_2_ agonist toxicity.

## Abbreviations

α_2_AR: Alpha-2 adrenergic receptor
IR: Imidazoline receptor
MOUD: Medications for opioid use disorder
OUD: Opioid use disorder
PWUD: People who use drugs
